# Milligan–Morgan hemorrhoidectomy vs. hemorrhoid artery ligation and recto-anal repair: a comparative study

**DOI:** 10.1186/s12893-022-01861-z

**Published:** 2022-12-06

**Authors:** Dimitrios Symeonidis, Michail Spyridakis, Dimitrios Zacharoulis, George Tzovaras, Athina A. Samara, Alexandros Valaroutsos, Alexandros Diamantis, Konstantinos Tepetes

**Affiliations:** Department of Surgery, University Hospital of Larissa, Mezourlo, 41110 Larissa, Greece

**Keywords:** Hemorrhoids, Milligan–Morgan hemorrhoidectomy, Hemorrhoidal artery ligation, Rectoanal repair

## Abstract

**Background:**

Several surgical techniques for the treatment of hemorrhoidal disease (HD) have been proposed. However, the selection of the most proper technique for each individual case scenario is still a matter of debate. The purpose of the present study was to compare the Milligan–Morgan (MM) hemorrhoidectomy and the hemorrhoidal artery ligation and rectoanal repair (HAL–RAR) technique.

**Methods:**

A retrospective analysis of the prospectively collected database of patients submitted to HD surgery in our department was conducted. Patients were divided into two groups, the MM group and the HAL–RAR group. Primary end points were recurrence rates and patients’ satisfaction rates. The Unpaired t test was used to compare numerical variables while the x^2^ test for categorical variables.

**Results:**

A total of 124 patients were identified, submitted either to HAL–RAR or MM hemorrhoidectomy. Eight (8) patients were lost to follow up and were excluded from the analysis. Of the remaining 116 patients, 69 patients (54 males and 15 females–male / female ratio: 3.6) with a median age of 47 years old (range 18–69) were included in the HAL–RAR group while 47 patients (40 males and 7 females–male / female ratio: 5.7) with a median age of 52 years old (range 32–71) comprised the MM group. At a median follow up of 41 months (minimum 24 months–maximum 72 months), we recorded 20 recurrences (28.9%) in the HAL–RAR group and 9 recurrences in the MM group (19.1%) (p 0.229). The mean time from the procedure to the recurrence was 14.1 ± 9.74 months in the HAL–RAR group and 21 ± 13.34 months in the MM group. Patients with itching, pain or discomfort as the presenting symptoms of HD experienced statistically significantly lower recurrences (p 0.0354) and reported statistically significantly better satisfaction rates (6.72 ± 2.15 vs. 8.11 ± 1.99—p 0.0111) when submitted to MM. In the subgroup of patients with bleeding as the presenting symptom, patients satisfaction rates were significantly better (8.59 ± 1.88 vs. 6.45 ± 2.70—p 0.0013) in the HAL–RAR group.

**Conclusions:**

In patients with pain, itching or discomfort as the presenting symptoms of HD, MM was associated with less recurrences and better patients satisfaction rates compared to HAL–RAR. In patients with bleeding as the main presenting symptom of HD, HAL–RAR was associated with better patients’ satisfaction rates and similar recurrence rates compared to MM.

## Introduction

Hemorrhoidal disease (HD) is the most common pathology of the anorectal region, affecting countless of patients worldwide [1]. So far, no single theory can comprehensively explain the pathophysiology of the disease [2]. Varicose veins, within the anal canal, have been implemented, but the theory involving structural changes of the supporting connective tissue of the area, which causes mucosal prolapse, seems to be more widely accepted [3, 4]. Regardless of the actual etiology, the consequences of the disease on the physiology of the anal canal are adequately documented, including increased resting anal pressures, lower rectal compliance, and excessive perineal descent [5, 6]. In regards to the clinical presentation, HD usually presents as painless rectal bleeding associated with bowel movements [7]. Prolapsing hemorrhoids may cause itching or discomfort in the perianal area due to the increased mucous secretion or soiling. Uncomplicated internal hemorrhoids are usually painless. If pain is present, other painful conditions of the perianal region such as fissure, abscess or even an anorectal neoplasm should be excluded [8].

Despite its notable limitations, the classic Goligher classification, which is based solely on the degree and the characteristics of the prolapse, is still the most widely used grading system for internal hemorrhoids [9]. First-line treatment options include dietary modifications with adequate intake of fiber and oral fluids, lifestyle changes, and sitz baths [10]. Outpatient treatments such as sclerotherapy and rubber band ligation should be used for the treatment of I, II, and III-degree HD in cases where conservative treatment fails [10, 11]. In general, the efficiency of these office based procedures in treating HD symptoms is increasingly appreciated [11]. A recent multicenter phase II trial reported pretty favorable results, in terms of efficacy and safety, following the use of sclerotherapy with 3% polidocanol foam in patients with bleeding second degree HD [12].

Finally, the surgical excision of hemorrhoids, in the form of either the Milligan-Morgan (MM) or the Ferguson procedure, remains a very effective approach for patients who fail or cannot tolerate office-based procedures, patients with grade III or IV HD, or patients with substantial concomitant skin tags [10, 11]. During the last 20 years, non-excisional surgical procedures for the treatment of HD such as the Doppler-guided hemorrhoidal artery ligation with or without mucopexy have been proposed as an alternative to traditional surgery [13]. Compared to the more invasive surgical techniques hemorrhoidal artery ligation seems to be associated with shorter operating time, less postoperative complications, and notably decreased postoperative pain [11, 14].

However, apart from the suture ligation of the hemorrhoidal arteries, other techniques have been also used to achieve the same local effect such as the hemorrhoid laser procedure (HeLP) [15]. This technique uses the selective shrinking and coagulating effects of laser energy on vessels to achieve hemorrhoidal dearterialization. Studies have confirmed the efficacy of the HeLP procedure in treating symptomatic HD [15, 16]. Similar to hemorrhoid artery ligation, mucopexy can be added to the HeLP procedure, in the so called HeLPexx alternative of the procedure, to address symptomatic mucosal prolapse, which may compromise the effectiveness of the HeLP procedure in patients with HD of higher degree [17].

In general, HD, with the exception of cases of severe bleeding and some rare disease associated complications such as gangrene is a condition which mainly affects the quality of patients’ life. Within this framework, the post treatment improvement of the quality of life scores and the increased patient satisfaction rates are designated as significant end points in regards to the long term effectiveness of each treatment approach. So far, even the most recent guidelines on the management of HD are almost consistently stage oriented [10, 11, 13]. The problem is that patients’ symptoms do not always correlate directly with the stage of HD [10]. Therefore, a redefinition of the indications of each operative approach, taking into account their effectiveness in alleviating certain HD symptoms appears justified. The purpose of the present study was to evaluate and compare two, well established, surgical techniques for the treatment of HD i.e. the Milligan–Morgan (MM) hemorrhoidectomy and the hemorrhoidal artery ligation and rectoanal repair (HAL–RAR) taking into account the presenting symptoms of HD. We looked primary for recurrences while patients’ satisfaction rates following the procedure were also to be assessed.

## Methods

Internal board approval and ethics committee (University Hospital of Larissa ethics committee) permission had been granted prior to the initiation of the present study. A retrospective analysis of the prospectively collected database of patients submitted to HD surgery in our department, from March 2013 to March 2018, was conducted. Indications for surgery were the following: persistent symptoms of HD after a failed trial of conservative treatment and sessions of non-surgical, office based procedures, symptomatic grade III and IV HD and severe, life threatening bleeding associated with HD [10, 11]. Apart from the indications for surgery, inclusion criteria for the present study were the following: age between 18 and 75 years, eligibility for elective surgery, and absence of major comorbidities i.e. American Society of Anesthesiologists score I or II [18]. Exclusion criteria were the following: previous anorectal surgery, associated anorectal disease such as anal fissure, fistula, abscess, rectal prolapse and fecal incontinence, American Society of Anesthesiologists score ≥ 3, other causes of chronic pain and mental illness.

In our department, we provide outpatient services and consultation, designated in anorectal disorders, 2 days per week (Outpatient clinic A and Outpatient clinic B) with distinct in regards to the offered surgical technique, when needed, treatment protocols. More specifically, patients, candidates for surgery, out of outpatient clinic A are submitted to MM hemorrhoidectomy while surgical candidates, out of outpatient clinic B, are offered the HAL–RAR procedure. For new patients, appointment scheduling, upon patient’s request, is done randomly, by the setting’s centralized appointment system, in either outpatient clinic A or B. Review appointments are scheduled, internally, to future, empty slots of the same outpatient clinic. There are six attending consultants, with years of experience in HD surgery, performing proficiently both of the procedures, responsible for these two clinics assigned in a weekly, rolling rota fashion. This setting’s policy actually divided patients into two groups i.e. the MM Group treated with MM hemorrhoidectomy and the HAL–RAR group treated with HAL–RAR procedure.

**Fig. 1:**
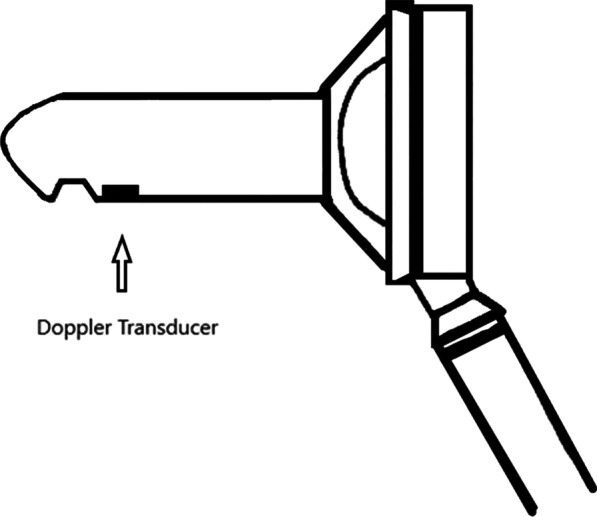
Proctoscope equipped with a Doppler transducer

Both techniques were performed, under the same principles by the six involved surgeons, either under spinal or general anesthesia. A detailed preoperative anesthetic evaluation and consultation was performed but patient preferences played an important role in the final decision regarding the type of anesthesia used. All patients had a rectal sodium phosphate enema, approximately 2 h, prior to the procedure. Antibiotic prophylaxis consisted of a single dose of a second generation cephalosporin administered intra-operatively. Only patients who required a Foley catheter placement received an additional dose of antibiotics. In regards to the HAL–RAR technique, with the patient in a lithotomy position a specially designed proctoscope (Fig. 1) equipped with a Doppler transducer was introduced into the anal canal. A typical HAL–RAR procedure was carried out under the principles previously described by Hoyuela et al. [19]. The procedure did not involve the removal of any hemorrhoidal tissue in all cases. In the vast majority of the cases hemostasis was adequate following the conclusion of the procedure. However, we chose placing a swab soaked with lidocaine gel 2% within the anal canal in all patients because our goal was to maintain the same operative principles irrespective of the individual case scenario. A classic open hemorrhoidectomy, as described by Milligan and Morgan, was the other operative technique [20]. The resulting wounds were left open and the anal canal was, similarly to the HAL–RAR procedure, plugged with a swab soaked with lidocaine gel 2%.

Postoperatively, the administration of prophylactic doses of low molecular weight heparin and proton pump inhibitors was common in all patients. A standard postoperative analgesic protocol was followed with oral paracetamol 500 mg every 6 h, and intravenous parecoxib sodium 40 mg twice a day. The standard analgesic protocol provided satisfactory pain control in the majority of patients. However, in 14 patients (4 patients from the HAL–RAR group and 10 patients from the MM group) additional opioid analgesics (50 mg of oral tramadol every six hours for a total of 48 h) were administered to achieve optimal pain control. The removal of the anal plug was conducted routinely 8 h after the procedure or sooner if the patients had a bowel movement prior to this point. Upon discharge, patients were given specific instructions in regards to the pain management, the return to the normal activity and how to deal with the wound in the cases where an excisional hemorrhoidectomy was undertaken. More specifically, patients were advised to use oral paracetamol combined or not with ibuprofen depending on the severity of pain, a mild laxative and a gauze pad in contact with the anal wound during the first days after the procedure.

In general, with the exception of severe bleeders, we initially treat patients with grade I and II HD, mainly conservative, with lifestyle and dietary modifications such as increasing oral fluid and fiber intake, reducing consumption of fat, avoiding excessive straining, and regular aerobic exercise. This conservative strategy is often supplemented with topical treatments containing corticosteroids, local anesthetics or anti-inflammatory agents. The duration of the conservative treatment varies depending on the effectiveness of the approach. As soon as a notable improvement on patients’ symptoms is observed, due to these lifestyle and dietary modifications, the length of this period is further prolonged, even up to the permanent deferral of any additional interventions. Although the majority of patients respond relatively well to these noninvasive treatments, there are indeed patients who fail to see their symptoms resolve. In these patients, office based procedures, mainly in the form of rubber band ligation are employed by the attending, at the outpatient clinic, consultant. Usually, up to three rubber bands are placed above the dentate line in a single session while multiple sessions might be required. In general, rubber band ligation is a well-tolerated procedure. Only a small minority of patients reported exacerbation of their “HD related” pain after this office based procedure which is usually sufficiently addressed by the use of over the counter painkillers alone. There were no recorded major complications such as major bleeding or local sepsis related to this minimally invasive approach.

A follow up was scheduled 2 weeks after the surgical intervention, as outpatients, in order to assess the immediate results of the operation. They were also contacted, at the time of data collection, for the preparation of this paper (March 2020), by phone interview, in order to update follow-up data regarding the long-term results, especially recurrences, and if that was the case the time interval between procedure and the recurrence was assessed, as well. In addition, patients’ were asked to give straightforward answers to two questions aiming to elicit the single, most annoying and/or alarming, presenting symptom and to assess the satisfaction rates following the procedure (Table 1). We chose using a non-standardized scale in order to assess patients’ satisfaction rates aiming to simplify the data collection process and the analysis of the results. According to the study design, the patients follow up was conducted by phone interview and the use of a properly validated questionnaire would be a rather complicated process.

**Table 1:** The questions asked during the phone follow up

Questions
1. Which one of the following two, Bleeding (A) or Pain / Itching / Discomfort (B) best describes the reason that made you seek surgical consultation about your hemorrhoidal disease problem?
2. In a scale of 1 (worse) to 10 (best), how satisfied are you from your hemorrhoidal disease surgery?

### Statistical analysis

The Graphpad^®^ software was used for the statistical analysis. The Unpaired t test was used to compare the differences between the two groups of the study in regards to numerical variables such as mean satisfaction rates. On the other hand, the Chi square test (x^2^) was used to compare differences in the two groups in regards to categorical variables such as sex (male / females) or recurrences. The results are expressed as respective p values. A result was considered statistically significant when p value was < 0.05.

## Results

A retrospective study of our prospectively collected database of patient submitted to either MM hemorhoidectomy or HAL–RAR in our department was conducted. A total of 124 patients were identified, submitted either to HAL–RAR or MM hemorrhoidectomy. Eight (8) patients were lost to follow up (5 patients submitted to HAL—RAR and 3 patients submitted to MM) and were excluded from the analysis. Of the remaining 116 patients (94 males and 22 females–male / female ratio: 4.2) with a median age of 48 years old (range 18–71), 69 patients (54 males and 15 females–male / female ratio: 3.6) with a median age of 47 year old (range 18 to 69) were included in the HAL–RAR group while 47 patients (40 males and 7 females–male / female ratio: 5.7) with a median age of 52 year old (range 32 to 71) comprised the MM group. In the HAL–RAR group, six (6) patients had II degree HD, thirty-five (35) patients had III degree HD and twenty-eight (28) patients had IV degree HD (Mean: 3.32 ± 0.63). The distribution of the HD stage in the MM group was as follow: nine (9) patients—degree II, twenty-two (22) patients—degree III and sixteen (16) patients—degree IV (Mean: 3.15 ± 0.72) (Table 2). The two groups were similar regarding the stage of the disease and the basic patients’ demographics (Table 3).

**Table 2:** The Goligher classification for hemorrhoidal disease

Degree	Description
I	Bleeding without prolapse
II	Hemorrhoids prolapse through the anus on straining but reduce spontaneously
III	Hemorrhoids prolapse through the anus on straining and require manual replacement
IV	Irreducible prolapsing hemorrhoids, acutely thrombosed or incarcerated hemorrhoids

**Table 3:** Patients sample characteristics and comparison of the general clinical data of the two groups of the study (HAL–RAR and MM)

	HAL–RAR	MM	x^2^ / t test	p value
Male / Female (n)	54 / 15	40 / 7	0.8524	.355862
Median age (range)	47 (18–69)	52 (32–71)	− 0.83065	.204263
Median BMI (range)	27 (21–37)	26 (20–39)	− 1.05099	.295505
ASA score I / II / III (n)	30 / 32 / 7	17 / 21 / 9	2.0293	.362521
Median procedure duration (range)	40 min (25–60)	45 min (35–75)	− 2.43503	.01644
Median Length of hospital stay (range)	1 day (1–2)	1 day (1–4)	1.89455	.060642
Degree I / II / III / IV (n)	0 / 6 / 35 / 28	0 / 9 / 22 / 16	2.7647	.250992
Spinal / General Anesthesia (n)	53 / 16	36 / 11	0.0007	.978454

Data are presented as number of patients (n) or median values (range). The unpaired t test was used to compare numerical variables while the Chi square test (x^2^) was used in the case of categorical variables

*HAL–RAR* Hemorrhoidal Artery Ligation and Rectoanal Repair, *MM* Milligan Morgan hemorrhoidectomy, *ASA score* American Society of Anesthesiologists score, *X*^*2*^ Chi square test, *t test* Unpaired t test

As patients’ preferences played a key role in the decision regarding the type of anesthesia, the majority of patients i.e. 89 patients had spinal anesthesia, while only 27 patients were operated under general anesthesia. Among patients submitted to spinal anesthesia, we recorded 4 cases of post spinal puncture headache (3 patients from the HAL–RAR group and 1 patient from the MM group) that resolved with symptomatic treatment alone. Urinary retention was a relatively common immediate complication postoperatively. Eleven (11) patients of the HAL–RAR group (all among those submitted to spinal anesthesia) and eight (8) patients of the MM group (6 submitted to spinal anesthesia and 2 patients to general anesthesia) required a short term bladder catheterization. In regards to the short term complications of the surgical techniques, we recorded 2 cases of clinically significant postoperative bleeding (diagnosed on the day of the operation in the first patient and on the 1st postoperative day in the second patient) in the HAL–RAR group and 5 cases in the MM group (three cases were diagnosed on the day of the operation, one on the 1st postoperative day and the last one on the 3rd postoperative day). In, one patient of the latter group the bleeding was too severe to be controlled by local conservative measures such as applying pressure with a gauze or tamponade the bleed with a vaseline or hemostatic gauze inserted within the anal canal and required reoperation where a suture ligation of the bleeding vessel was performed.

Interestingly, there were no recorded septic complications or delayed bleeding cases in any of the patients included in the study. Postoperative pain control after discharge proved adequate with the standard prescribed protocol i.e. paracetamol and ibuprofen. Only two patients (one patient from the HAL – RAR group on postoperative day 3 and one patient from the MM group on postoperative day 5) required readmission because of severe, not controlled with the oral painkillers, pain that required the administration of intravenous opioids. In regards to the long term complications, one patient from the MM group developed a moderate stenosis of the anal canal, 8 months after the operation, which was however sufficiently addressed with manual anal dilations alone on an outpatient basis. Although we did not use specially designed questionnaires to assess the true incidence of incontinence, however none of the patients in the study reported symptoms consistent with clinically significant incontinence.

At a median follow up of 41 months (range: minimum 24 months–maximum 72 months), we recorded 20 recurrences (28.9%) in the HAL–RAR group and 9 recurrences in the MM group (19.1%) (p = 0.229). The mean time from the procedure to the recurrence was 14.1 ± 9.74 months for the patients with recurrence in the HAL–RAR group and 21 ± 13.34 months for the patients with recurrence in the MM group. In the subgroup of patients with itching, pain or discomfort as the presenting symptom recurrence rates were statistically significantly lower (P 0.0354) in patients submitted to MM compared to HAL–RAR. Regarding recurrences, there was no differences recorded between the MM and the HAL–RAR group in the subgroup of patients with bleeding as the presenting symptom (Table 4).

**Table 4:** Comparison, using the Chi square test (x^2^), of the two groups of the study, according to the presenting symptom, in regard to recurrences

Symptoms	Degree	HAL–RAR		MM		x^2^	p value
		No of patients	Recurrences	No of patients	Recurrences		
Bleeding	II	1	–	4	1		
	III	18	2	8	2		
	IV	14	2	8	1		
	Total	33	4	20	4	0.6032	0.437
Discomfort / Itching / Pain	II	5	4	5	1		
	III	17	6	14	2		
	IV	14	6	8	2		
	Total	36	16	27	5	4.6667	.030754

*HAL–RAR* Hemorrhoidal Artery Ligation and Rectoanal Repair, *MM* Milligan Morgan hemorrhoidectomy, *X*^*2*^ Chi square test

In the present study, we assessed patient satisfaction scores following their HD surgery by asking patients, during the phone follow up interview, to rate, in a 1 (worst) to 10 (excellent) scale, their satisfaction out of their HD surgery. In the subgroup of patients with itching, pain or discomfort as the presenting symptoms of HD, patients’ satisfaction rates were significantly better in the MM group of the study (6.72 ± 2.15 for the HAL–RAR group vs. 8.11 ± 1.99 for the MM group—p = 0.0111). On the other hand, in the subgroup of patients with bleeding as the presenting symptom, patient satisfaction rates were significantly better in the HAL–RAR group of the study (8.59 ± 1.88 for the HAL–RAR group vs. 6.45 ± 2.70 for the MM group—p = 0.0013) (Table 5).

**Table 5:** Comparison, using the unpaired t test, of the Mean ± standard deviation (SD) patients’ satisfaction rates in the two groups of the study

Symptoms	Degree	HAL–RAR		MM		p value
		No of patients	Mean ± SD patient satisfaction rate	No of patients	Mean ± SD patient satisfaction rate	
Bleeding	II	1	10,0 ± 0	4	9 ± 1,41	
	III	18	8,11 ± 2,08	8	5,25 ± 2,38	
	IV	14	9,07 ± 1,59	8	6,38 ± 2,83	
	Total	33	8,59 ± 1,88	20	6,45 ± 2,70	0.0013
Discomfort / Itching / Pain	II	5	6,0 ± 2,45	5	9,2 ± 0,84	
	III	17	6,82 ± 2,48	14	7,5 ± 2,5	
	IV	14	6,86 ± 1,66	8	8,5 ± 0,93	
	Total	36	6,72 ± 2,15	27	8,11 ± 1,99	0.0111

*HAL–RAR* Hemorrhoidal Artery Ligation and Rectoanal Repair, *MM* Milligan Morgan hemorrhoidectomy, *SD* standard deviation

## Discussion

HD is the most common anorectal pathology with direct consequences on the quality of the patients’ life [10, 21]. The Goligher classification, published in 1980, is still the most used system for classifying internal hemorrhoids [9]. This classification, although widely accepted, has certain limitations. First, it does not take into account the possible coexistence of external HD component, as well. In this setting, the presence of thrombosis or the, associated with the chronic perianal inflammation, skin tags can be the prime source of symptoms. Second, it was developed during a time period with limited treatment options for HD. And third, there are notable differences, even between patients of the same HD stage, in regards to the degree of prolapse and the severity of symptoms. The need for more descriptive and analytical classification systems is obvious. In this direction, the BPRST classification, which has been recently proposed, takes into account parameters such as bleeding, prolapse, reduction, skin tags, and, thrombosis in order to classify HD [22]. Therefore, important HD characteristics, not properly acknowledged by the Goligher classification, are increasingly appreciated in the more recent reports [22, 23]. An approach of individualizing the treatment strategy according to the specific HD characteristics appears reasonable.

An ideal surgical treatment for HD should have the following characteristics: minimal postoperative pain allowing patients to return to their normal activities promptly and zero recurrence and complication rates. So far, no single proposed technique combines all of these characteristics. For instance, the classic excisional techniques appear to be superior in regards to recurrence rates at the cost, however, of increased postoperative pain [10]. On the other hand, the non-excisional surgical techniques have the advantages of less associated local trauma, minimal postoperative pain and more rapid recovery but seem to be associated with increased recurrence rates compared to the classic excisional techniques [10, 11, 21, 23]. In general, the assessment of the results of a certain surgical intervention for HD is a challenging procedure. The vast majority of HD patients seek surgical consultation due to symptoms affecting their quality of life. It is the relief of these symptoms the crucial post-treatment event that becomes synonymous with cure for these patients. We are probably missing important elements when we are assessing the results of a surgical intervention in an outpatient setting by physical examination alone. The assessment of patients’ satisfaction out of the procedure, which is directly related with the elimination of symptoms, can complement effectively the clinical evaluation process. Quality of life questionnaires have been used to objectify patients’ satisfaction following a certain intervention. However, the scores yielded out of these questionnaires, though of important value in the short term, are less representative of the specific treatment effect in the long term as contamination might ensue.

In the present study, we used our prospectively collected database of patients submitted to HD surgery in our department, either MM or THD–HAL, in order to compare the two techniques. We aimed for a minimum time to follow up of two years in order to more accurately assess recurrences. According to the results, the two techniques were not different in regards to the study end points. Interestingly, the subgroup analysis designated MM hemorrhoidectomy as superior to HAL–RAR in regards to recurrences and patients’ satisfaction rates in patients with pain, itching or discomfort as the presenting symptoms of HD. The fact that the MM hemorrhoidectomy addresses more effectively and in a more permanent manner, than the HAL–RAR technique, the associated prolapse might be responsible for this result. On the other hand, as the theory behind HLA–RAR is to reduce the inflow of blood into the hemorrhoidal venous plexus, could explain the documented, in the given patient sample, efficiency of the technique in patients with bleeding as the presenting symptom of HD.

The effectiveness of HAL–RAR as a valid treatment option of HD has been adequately established in the literature [19, 23]. It is now considered a safe and effective technique related to a high percentage of success, low complication, and recurrence rates [24]. However, there are patients that seem to benefit more out of this minimally invasive operative technique. Accurately defining this patient group could further highlight the advantages of the procedure. Aligned with the results of the present study but with relatively different symptom adjusted patient grouping, Scheyer et al. reported that HAL–RAR provided prolonged relief for patients with HD whose main symptoms are bleeding, pruritus and pain but not for patients with prolapse as the initial indication [25]. In addition, Lopez et al. in their prospective randomized trial comparing HAL–RAR with excisional hemorrhoidectomy reported that bleeding resolution was observed earlier in patients submitted to HAL–RAR than in patients submitted to excisional hemorrhoidectomy [26].

Although, we particularly aimed in eliminating bias, our study has several limitations. The retrospective nature of the study, the lack of proper randomization and the consequent selection bias should be kept in mind when interpreting the results. In addition, appropriate statistical matching was not used and this may have affected the results. The relatively small patient sample and the fact that we did not use a properly validated method in order to assess patients’ satisfaction following the procedure represent additional limitations. We have also included patients with grade II HD in the analysis; a patient group that less invasive office based procedures might represent the definite treatment. In support of this notion, the Hubble trial, a multicenter randomized control trial, that compared hemorrhoidal artery ligation with rubber band ligation for the management of symptomatic second-degree and third-degree HD reported that although hemorrhoidal artery ligation is more effective than a single session of rubber band ligation, If rubber band ligation is considered a course of repeated sessions, the procedures appear equally effective [27]. However, the present study highlights the facts that the main presenting symptom of HD should probably be included in the challenging equation of selecting of most proper surgical technique among surgical candidates for HD surgery. A prospective randomized trial, detached from the limitations of a retrospective analysis, is currently underway in our department aiming to provide more solid answers in regards to the most appropriate surgical technique.

In conclusion, in patients with pain, itching or discomfort as the presenting symptoms of HD, MM was associated with less recurrences and better patients satisfaction rates compared to HAL–RAR. In patients with bleeding as the main presenting symptom of HD, HAL–RAR was associated with better patients’ satisfaction rates and similar recurrence rates compared with MM.

## Data Availability

The datasets analysed during the current study are not publicly available due patients’ privacy but are available from the corresponding author on reasonable request.
